# Determinants of circulating calcitonin value: analysis of thyroid features, demographic data, anthropometric characteristics, comorbidities, medications, and smoking habits in a population with histological full exclusion of medullary thyroid carcinoma

**DOI:** 10.3389/fonc.2024.1278816

**Published:** 2024-01-29

**Authors:** Pierpaolo Trimboli, Giuseppe Peloni, Dorotea Confalonieri, Elena Gamarra, Tommaso Piticchio, Francesco Frasca, Petra Makovac, Arnoldo Piccardo, Lorenzo Ruinelli

**Affiliations:** ^1^ Servizio di Endocrinologia e Diabetologia, Ospedale Regionale di Lugano, Ente Ospedaliero Cantonale (EOC), Lugano, Switzerland; ^2^ Facoltà di Scienze Biomediche, Università della Svizzera Italiana (USI), Lugano, Switzerland; ^3^ Servizio di Chirurgia, Ospedale Regionale di Mendrisio, Ente Ospedaliero Cantonale (EOC), Lugano, Switzerland; ^4^ Endocrinology Section, Department of Clinical and Experimental Medicine, Garibaldi Nesima Hospital, University of Catania, Catania, Italy; ^5^ Department of Nuclear Medicine, E.O. "Ospedali Galliera", Genoa, Italy; ^6^ Team Data Science & Research, Area ICT, Ente Ospedaliero Cantonale, Bellinzona, Switzerland; ^7^ Clinical Trial Unit, Ente Ospedaliero Cantonale (EOC), Bellinzona, Switzerland

**Keywords:** calcitonin, determinants, thyroid nodules, medullary thyroid carcinoma, anthropometric characteristics, drugs, interference

## Abstract

**Objective:**

Calcitonin (Ctn) measurement is crucial for the early diagnosis of medullary thyroid carcinoma (MTC). However, Ctn levels can be skewed/elevated due to other reasons, and the Ctn upper reference value remains controversial. In this field, studies have heterogeneous settings, published data are controversial, and no evidence has been achieved. The study’s aim was to evaluate all previously investigated Ctn determinants in a population with histological exclusion of MTC.

**Methods:**

The institutional records from 2010 to 2022 were reviewed to select patients with thyroid nodules who had undergone total thyroidectomy with histological exclusion of MTC and who had tested for Ctn just before surgery. Thyroid features, demographic and anthropometric data, comorbidities, medications, and lifestyle information were collected. Univariate and multivariate analyses were performed.

**Results:**

A total of 127 cases were included. The median age for thyroidectomy was 51 years. Median Ctn was 1.04 pg/mL (interquartile range (IQR) 1.04–2.77), with two cases having values above 10 pg/mL. In univariate analysis, Ctn was correlated with gender (p < 0.001), body weight (p = 0.016), height (p = 0.031), body surface area (p = 0.016), thyroid size (p = 0.03), thyroglobulin (p < 0.001), and chronic kidney disease (p < 0.001). After multivariate analysis, the model with the highest accuracy included gender, chronic kidney disease, and thyroid-stimulating hormone (TSH) with an adjusted R-squared of 0.4.

**Conclusions:**

This study demonstrates, in a population histologically proven as MTC-free, that the Ctn value is mainly influenced by gender, anthropometric/thyroid features, and chronic kidney disease, with the further impact of TSH.

## Introduction

Medullary thyroid carcinoma (MTC) originates from thyroid C cells and can be associated with poor/fatal prognosis depending on its stage at diagnosis and initial treatment (1). Even if calcitonin (Ctn) is well recognized as essential to diagnose MTC (2), its routine measurement in patients with thyroid nodules is not universally accepted for several reasons. First, MTC is an infrequent cancer. Second, the Ctn value may depend on the assay and other individual factors (3). Third, although it is proven that the higher the Ctn value, the higher the likelihood to detect MTC, we cannot use a fixed Ctn threshold to exclude it (4, 5). On the one hand, Ctn levels >100 pg/mL are virtually diagnostic for MTC, and Ctn below the upper reference limit virtually excludes it. On the other hand, Ctn between the upper reference limit and 100 pg/mL (indeterminate Ctn) merits to be carefully interpreted (4–6). Moreover, although the evidence-based data indicate that 0.11%–0.85% of patients with thyroid nodules can harbor MTC (7), no univocal recommendation is available from international societies about using or not Ctn as a screening test in patients with thyroid nodules (4, 5, 8, 9).

Among the above issues, the matter of factors influencing Ctn value is still open. In addition to those factors increasing Ctn levels by physio-pathological mechanisms, such as chronic kidney disease (CKD) and bacterial infection, the influence of intrinsic patient characteristics has been also suggested as a determinant of Ctn upper reference value. Particularly, men should have a higher Ctn than women, and it has been hypothesized that Ctn value changes according to thyroid size, thyroid-stimulating hormone (TSH) value, thyroid autoimmunity, cigarette smoking habits, alcohol consumption, and use of proton pump inhibitors (PPIs). Nevertheless, these findings have not achieved full consideration for several reasons. Indeed, these data derive from unselected populations and are somewhat discordant among studies (10–19). Also, a major concern about these findings is that the studies did not fully exclude the presence of MTC. Indeed, inadequate/suboptimal standard of reference has been used since patients were defined as MTC-free according to fine-needle aspiration cytology (FNAC) (20, 21) or even palpation (10). In addition, thyroid volume was always calculated on ultrasound, which represents an estimation bias. Thus, due to the fragility of data, the interferents or the determinants of Ctn values are still under debate.

With these premises, the present study was conceived to achieve higher evidence about the role of thyroid features (i.e., thyroid size, hormones, and autoimmunity), demographic data (i.e., gender and age), anthropometric characteristics (i.e., weight and body measurements), comorbidities (i.e., CKD), medications (i.e., PPI), and smoking as determinants of Ctn value. Importantly, to perform a full analysis, differently from all the previous studies on this topic, here, only patients who had undergone total thyroidectomy with histologically proven exclusion of MTC were included.

## Methods

### Study design and data extraction

According to the study conceptualization, the institutional database was screened to find the specific setting of patients with thyroid nodules who had undergone total thyroidectomy and who had tested for Ctn just before surgery. In fact, patients undergoing thyroid surgery at our institution are generally tested for Ctn just before the operation. The study period was set from January 2010 to October 2022. Later, patients with active bacterial infection or who refused to participate in the study were excluded. Then, the following data were extracted from records of all included cases: gender, age on thyroidectomy, histological diagnosis, thyroid size expressed in grams of weight recorded during histological examination, value of Ctn, thyroid hormones, thyroglobulin (Tg), anti-thyroid antibodies (T-Ab) collected just before surgery, comorbidities with potential to influence Ctn (i.e., CKD), medications (i.e., PPI), and smoking (i.e., current smoker, ex-smoker, and non-smoker).

### Reference standard for excluding MTC

The histological diagnosis was the gold standard for diagnosis, allowing us to include in the study only patients without MTC and C-cell hyperplasia.

### Laboratory tests

The measurement of Ctn was performed on the Immulite 2000 XPi platform (Siemens Healthcare Diagnostics, Malvern, PA, USA) until February 2019 and on the Cobas 8000 platform (Roche Diagnostics, Basel, Switzerland) since March 2019. Thyroid hormones and T-Ab were measured on the Immulite 2000 platform (Siemens Healthcare Diagnostics) until 2018 and Cobas 6800 (Roche Diagnostics) later.

### Ethics

The study was approved by the Tessin Cantonal Ethics Committee.

### Statistical analysis

Univariate analyses were performed for all parameters to assess their correlation with the Ctn value and their mutual relationship. The parameters herein included as potential Ctn determinants were considered as dichotomic and/or continuous variables when clinically appropriate. Dichotomic variables were expressed as positive/altered or negative/normal. Continuous variables were reported as median and interquartile range (IQR). The correlation between Ctn and continuous parameters was analyzed by Pearson’s test. Paired and unpaired data of continuous variables from two groups were compared using the Mann–Whitney test. Stepwise multivariate regression analysis was performed to evaluate which variables influence Ctn independently from the other ones. Statistical significance was set at p < 0.05.

## Results

### Patient flow and main data

According to the above selection criteria, 128 subjects were enrolled in the study, of whom one refused to be included. The 127 included cases (88 women) presented a median age on thyroidectomy of 51 years. Median Ctn was 1.04 pg/mL (min 0.35, max 18.72, IQR 1.04–2.77). Only two cases had Ctn above 10 pg/mL. Data of determinants included in the study were generally available in the series. No significant difference was found when comparing Ctn values recorded using the two different assays employed during the study period. Specifically, there were no significant differences between the two assays in terms of median and IQR of Ctn, and the two assays did not show different Ctn values according to the variables included in the present study. **Tables 1**, **2** illustrate the results of the analysis considering continuous and dichotomic variables, respectively.

**Table 1:** Analysis of correlation between all continuous variables and Ctn value.

Parameter	Cases with available data	Median value (IQR)	p
Age (years)	127	51.0 (40.5–60.0)	0.102
Body weight (kg)	119	68.0 (58.0–82.5)	0.016
Height (cm)	113	166.0 (159.0–172.0)	0.031
BMI (kg/m^2^)	113	24.5 (22.2–28.8)	0.227
BSA (m^2^)	113	1.8 (1.6–2.0)	0.016
Thyroid weight (g)	120	27.1 (16.2–48.4)	0.026
TSH (mIU/L)	116	1.4 (0.5–2.1)	0.445
Free T3 (pmol/L)	62	5.1 (4.6–5.8)	0.190
Free T4 (pmol/L)	66	13.9 (10.5–16.1)	0.166
Thyroglobulin (μg/L)	94	33.8 (11.4–107.7)	<0.001

IQR, interquartile range; BMI, body mass index; BSA, body surface area; Ctn, calcitonin.

**Table 2:** Analysis of association between dichotomic variables and Ctn value.

Parameter	Cases with available data	Value	p
Gender	127	88 women	<0.001
Kidney function	123	4 with CKD	<0.001
PPI	124	11 users	0.21
Thyroglobulin	94	37 higher than normal reference	0.32
T-Abs	83	28 positives	0.73
Other thyroid cancer	127	77 with PTC	0.61
Smoking	107	36 current smokers	0.31

CKD, chronic kidney disease; PPI, proton pump inhibitor; T-Ab, anti-thyroid antibodies; PTC, papillary thyroid carcinoma; Ctn, calcitonin.

### Thyroid features (i.e., thyroid size, malignancy, hormones, and autoimmunity)

Thyroid weight recorded during histological examination was positively correlated with Ctn (p = 0.03). Thyroglobulin was significantly correlated with Ctn (p < 0.001). Thyroid hormones and T-Ab were not associated with Ctn. The histological diagnosis of differentiated thyroid carcinoma was not associated with Ctn.

### Demographic data

**Figure 1:**
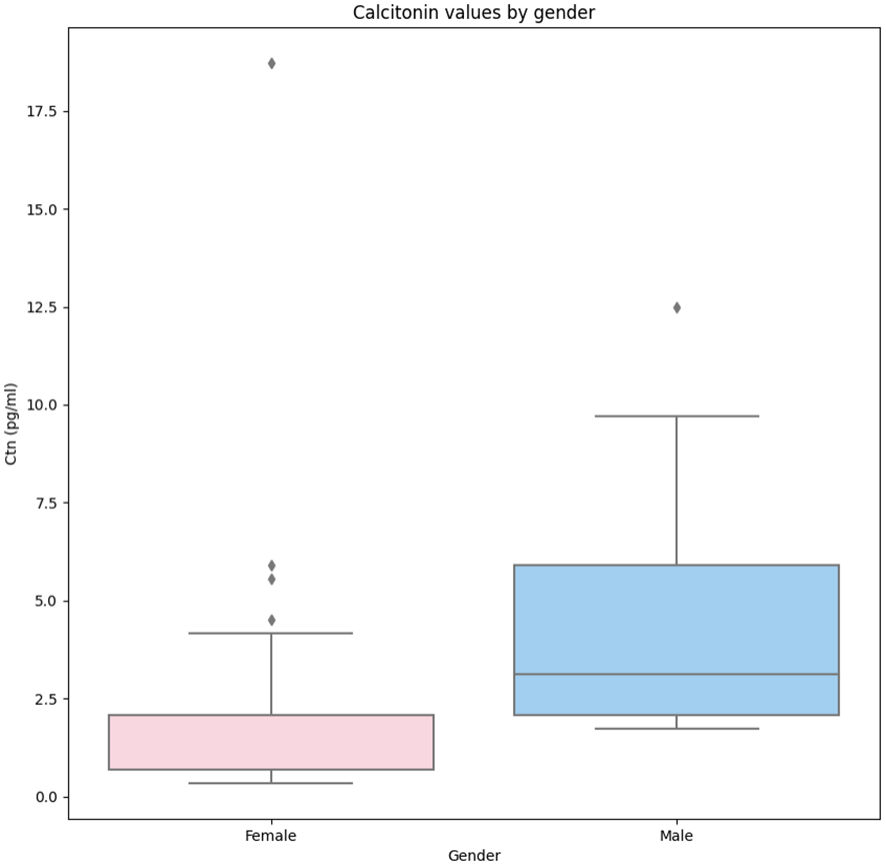
Comparison of Ctn values recorded in women and men enrolled in the present study. Legend: The box plots indicate the 50% value distribution and IQR. The points indicate the outlier values. IQR, interquartile range.

A significant association between gender and Ctn was observed (p < 0.001). As shown in **Figure 1**, when the Ctn value of men (3.12 pg/mL, IQR 2.08–5.89) was compared with that of women (2.08 pg/mL, IQR 0.69–2.08), a significant difference was found (p < 0.001). Age was not significantly associated with Ctn; the population was also analyzed according to age tertiles and quartiles, and no significant difference in Ctn values was observed among subgroups.

### Anthropometric characteristics

Body weight (p = 0.016), height (p = 0.031), and body surface area (BSA) (p = 0.016) were significantly correlated with Ctn. No correlation was found between BMI and Ctn.

### Comorbidities

The Ctn of patients diagnosed with CKD ranged from 0.35 to 18.72 pg/mL. The presence of CKD was significantly associated with Ctn (p < 0.001).

### Medications

There were 11 on PPI. The Ctn value of this subgroup was not significantly different from the other ones (p = 0.21).

### Lifestyle

As detailed above, patients were defined as current smoker (n = 36), ex-smoker (n = 9), or non-smoker (n = 62). When considering current smoking as a dichotomic parameter, there was no association with Ctn. No significant association was also observed when the three subgroups were compared to each other in multiple combinations. The Ctn of current smokers (2.08 pg/mL, IQR 0.95–3.12) was not significantly (p = 0.48) different from that of the remaining patients (2.08 pg/mL, IQR 1.56–2.43). No data about alcohol consumption were available in the database.

### Comparison between women and men

**Figure 2:**
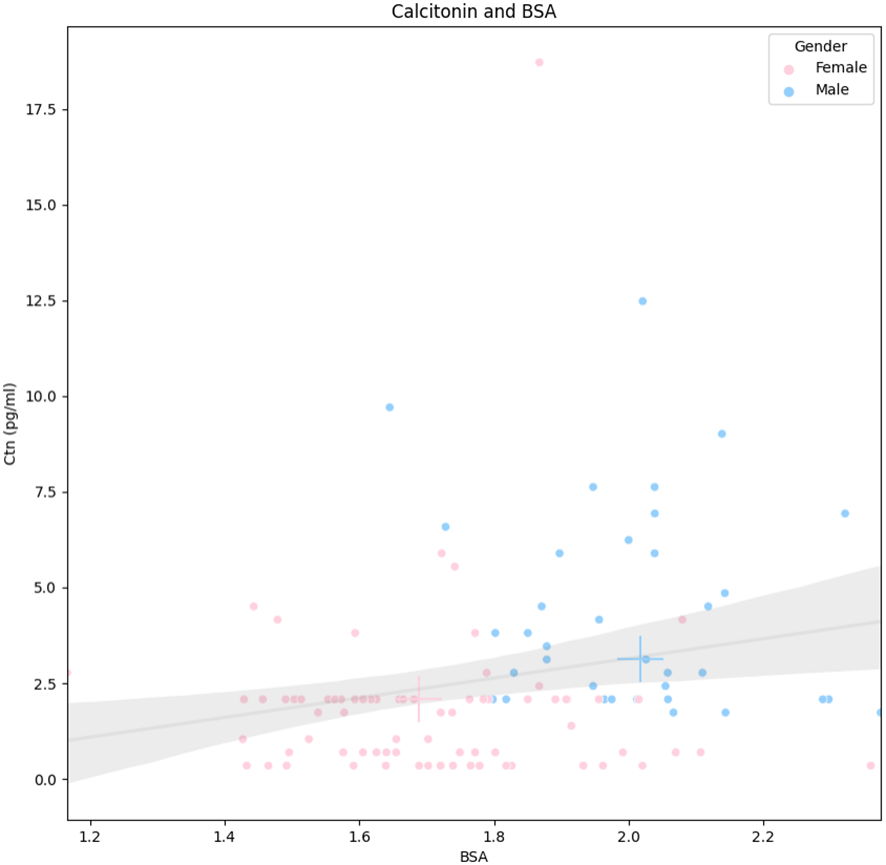
Correlation between Ctn and BSA according to gender. Legend: Continuous line represents the linear regression, and the gray area is its standard deviation (whole population). Pink and blue crosses indicate median values in women and men, respectively. Ctn, calcitonin; BSA, body surface area.

As mentioned above, the Ctn of men was significantly higher than that of women. In addition, men showed significantly higher body weight, height, and BSA (p < 0.001) than women. The other parameters were not significantly different between the two groups. According to these results, the relationship between Ctn and BSA was further explored. **Figure 2** illustrates the correlation between the two parameters according to gender.

### Multivariate analysis

The backward and forward linear regression estimates provided the same results. The model with the highest accuracy included gender, CKD, and TSH. Women had significantly lower Ctn levels compared to men (on average −2.55 pg/mL, p < 0.001). CKD was associated with higher Ctn levels (on average +7.24 pg/mL compared to patients without kidney failure, p < 0.001). Regarding TSH, on average, an increase of 1 mIU/L in the TSH level was associated with a reduction in the Ctn level of 0.15 pg/mL (p-value <0.001). The adjusted R-squared shows that the three predictors included account for approximately 40% of the variance in the Ctn level.

## Discussion

Due to the suboptimal accuracy of cytology and ultrasound (20–22), the diagnosis of MTC remains challenging. Routine Ctn measurement in patients diagnosed with nodular thyroid disease is still controversial in the literature and has not been fully accepted due to several reasons. Among them, individual determinants hold a main role, and Ctn stimulation tests have been proposed to overcome the limitations of basal Ctn. Nevertheless, although the Ctn stimulation test was conceived to differentiate thyroid causes of elevated Ctn from non-thyroid sources (23), it requires careful procedure under medical control, and no high evidence of fixed cut-off is available (2). Therefore, the potential impact of several determinants on circulating basal Ctn may hold a crucial role. However, whether a patient’s specific upper reference values have to be used in clinical practice has been largely debated but remains an open matter. **Table 3** illustrates the main features of the previously published studies in this thyroid field. Some authors found that Ctn value can be influenced by patient and/or thyroid features, comorbidities/medications, and smoking, while these results were not confirmed by others. Also, each study analyzed only a part of potential interfering factors. In addition, highly important, these studies presented a quite heterogeneous series of patients (i.e., with a thyroid nodule, without nodule according to palpation; selected for biopsy; defined as healthy during population screening programs; with type 2 diabetes and high cardiovascular risk) without full proof of the absence of MTC in almost all studies. In some cases, the enrolled patients did not present thyroid nodules, and from a clinical point of view, these are useless data because we should test for Ctn in patients with thyroid nodules only. Thus, the data of each study could not be interpreted beyond the simple statistical figure. All in all, before the present one, there was no study 1) evaluating all the potential influencing factors investigated in the literature and 2) including only patients who have undergone total thyroidectomy. The latter has to be taken into account when looking at the data presented in the other studies because there were some patients with mild-to-moderate Ctn increase without histological exclusion of MTC. We should ask ourselves whether we can consider those cases MTC-free, especially in view of the recent high-evidence data reporting the suboptimal accuracy of FNAC and ultrasound in detecting this cancer (20–22). Until proven otherwise, those patients may be affected by small/occult MTC or C-cell hyperplasia.

**Table 3:** Characteristic and major results of articles evaluating Ctn determinants.

First author, year [ref]	Setting	Cases	Ctn assay	Determinants analyzed	Results
d’Herbomez, 2007 (10)	Subjects with no history of thyroid disease, no goiter at physical examination, no medication known to influence thyroid tests, with normal calcium levels. Ctn was measured with five assays	287 (142 men, 145 women)	Five different immunoassays: A) immunoradiometric assay–human calcitonin (IRMAhCT) (CisBio International); B) CT on Advantage (Nichols Institute, San Clemente, CA, USA); C) CT on Immulite 2000 (DPC, La Garenne-Colombes, France); D) Calcitonin-ELISA 7024 (Biomerica, Newport Beach, CA, USA); E) CT-USA-IRMA, KIP0429 (Biosource, Nivelles, Belgium)	Age, gender, BMI, smoking	Age was correlated with Ctn in four assays in male smokers. BMI was correlated with Ctn with four assays. For three assays, positive smoking status increased the interpretation threshold
Giovanella, 2012 (12)	Thyroid-healthy volunteers enrolled in local thyroid screening program. Ctn was measured with three different assays	519 (246 men, 273 women)	Three different immunoassays: A) immunoradiometric (IRMA) method ELSA^®^ -hCT (CIS Bio, Gif sur Yvette, France) [CIS]; B) chemiluminescent immunometric assay Immulite^®^ 2000 XPi (Siemens Diagnostics, Milan, Italy) [Immulite]; C) immunometric assay LIAISON^®^ CT-II (DiaSorin, Saluggia, Italy) [LIAISON]	Age, gender, smoking, and US-estimated thyroid volume	Men have higher Ctn than women. Thyroid volume is correlated with Ctn (but not for all Ctn assays)
Grani, 2012 (13)	Patients underwent FNAC and Ctn test	1,073 (180 men, 893 women)	Immunochemiluminometric assay	Age, T-Ab, FNAC	No correlation between Ctn and the included parameters
Rosario, 2013 (14)	Three groups: patients with thyroid nodules evaluated before thyroidectomy, patients without nodules, athyreotic PTC patients with distant metastases	1,137 (487 men, 650 women) divided into three unmatched groups	Immunochemiluminescence assay (Immulite, Diagnostic Products Corporation, Los Angeles, CA)	PTC, Hashimoto/T-Ab, smoking	No correlations between Ctn and the included parameters
Guesgen, 2013 (15)	Patients with autoimmune thyroiditis or nodular goiter or thyroid disease-free, using or not PPI	493 (406 men, 87 women) divided into five unmatched groups	Immunoradiometric assay (Calcitonin IRMA magnum, MEDIPAN)	Gender, autoimmune thyroiditis, nodular goiter, PPI	Men had significantly higher Ctn than women
Camacho, 2014 (16)	Normal individuals. Patients with autoimmune thyroiditis, nodular thyroid disease, differentiated thyroid cancer (free of disease), or chronic renal failure	178 normal individuals (47 men, 131 women). 205 with autoimmune thyroiditis (44 men, 161 women). 248 with nodular thyroid disease (22 men, 226 women). 80 with differentiated thyroid cancer (11 men and 69 women). 58 with chronic renal failure (25 men, 33 women).	Immunofluorometric assay	Gender, autoimmune thyroiditis, nodular thyroid disease, differentiated thyroid cancer, chronic renal failure	Almost all cases with nodular disease and autoimmune thyroiditis had Ctn below normal cut-off. Patients with chronic renal failure had Ctn above the cut-off of normality for men (≈4%) and for women (≈6%). Patients with differentiated thyroid cancer had normal Ctn.
Daniels, 2015 (17)	People with type 2 diabetes at cardiovascular risk	9,340 (6,003 men, 3,337 women)	Chemiluminescent immunometric assay (Siemens Healthcare Diagnostics, Malvern, PA, USA)	Gender, age, race, thyroid disease, PPI, thyroiditis, smoking, BMI, HbA1c, triglycerides, HDL, LDL, H2 receptor antagonists, eGFR, diabetes duration, β-blockers	Ctn was higher in men than women. Kidney function, smoking, and BMI were associated with higher Ctn. The data about smoking seemed to be influenced by gender
Song, 2021 (18)	Adults with or without thyroid disease and have undergone thyroid US during clinical checkup	10,566 (6,341 men, 4,225 women)	Immunoradiometric assay–human calcitonin (IRMA-hCT; CisBio International, Codolet, France)	Age, gender, BMI, smoking, kidney function, T-Ab	Men have higher Ctn than women. Smoking increases Ctn in men but not in women
Cvek, 2021 (19)	Patients with autoimmune thyroiditis compared to thyroid-healthy controls	467 (34 men, 433 women)	Immunoradiometric assay (IRMA) DIAsource ImmunoAssays (Louvain-la Neuve, Belgium, catalog number KIP0429)	Autoimmune thyroiditis, age, BSA	No influence of autoimmune thyroiditis on Ctn

BSA, body surface area; BMI, body mass index; eGFR, estimated glomerular filtration rate; FNAC, fine-needle aspiration; HbA1c, glycated hemoglobin; T-Ab, anti-thyroid antibodies; PPI, proton pump inhibitor; PTC, papillary thyroid carcinoma; US, ultrasound.

Here, we revised our institutional database with the aim of fully evaluating Ctn value in a setting of patients with complete absence of MTC or C-cell hyperplasia as demonstrated at histology after total thyroidectomy. This means that we could actually perform a full analysis of Ctn determinants in a population expected to have normal values. As a result of univariate analysis, Ctn was significantly higher in men than women and significantly correlated with individual anthropometric characteristics as well as thyroid size (and its surrogates), while according to multivariate analysis, gender, CKD, and TSH explained 40% of the Ctn variance. Moreover, men had significantly higher weight and BSA, the latter finding being the reason why anthropometric features did not influence Ctn levels in multivariate analysis. The robustness of these figures is given by the same result obtained with forward and backward linear regression as well as the ability of the model to explain a high percentage of Ctn value variance. The evidence achieved merits a careful clinical discussion.

The possibility of facing patients with Ctn skewed below the well-recognized diagnostic threshold of 100 pg/mL has represented a further brake on the implementation of routine Ctn in thyroid nodule patients. In fact, regardless of the lack of clear international recommendations in favor or against routine Ctn, how to manage patients with Ctn above the upper reference and below 100 pg/mL has still to be clarified.

The present data clearly show that, with the exclusion of CKD, the only parameters to be considered to influence Ctn are gender and anthropometric features.

Despite the findings observed in the previous studies that included heterogeneous series of patients, the role of other factors seems to be marginal or absent. In fact, since a strong correlation between gender and anthropometric features was found in our series, a selection bias could affect previous studies. Indeed, heterogeneous and discordant results observed among studies could be due to several factors: 1) male-to-female ratio; 2) age; 3) prevalence of goiter, autoimmune thyroiditis, or cancer; 4) body and thyroid measurements; 5) Ctn assay performance; 6) institutional management of thyroid nodule patients with or without adoption of routine Ctn test, with or without FNAC, and both indication for and interpretation of Ctn stimulation tests; 7) indication for surgery; 8) comorbidities and medications; 9) lifestyle; and others. Nevertheless, the present study cannot completely exclude an individual effect of T-Ab, smoking, other thyroid cancers, or others that might be taken into account for anecdotal cases. Regarding age, it is worth noting that no significant difference was observed among different ages; thus, the present data seem reliable independently of age. In contrast, it is necessary to take into account the role of medications and PPI first (24), which can influence Ctn levels. Regarding this specific point, only a minority of cases of our population used PPI, and this small frequency could not allow us to fully explore the PPI impact on Ctn. Therefore, gender and anthropometric characteristics (and thyroid size or its surrogate, such as Tg) can be considered in clinical practice to justify mildly elevated Ctn (i.e., >10 pg/mL). TSH is generally an expression of large goiter, and this can explain its role in the above multi-parameter model. Also, TSH and thyroid size depend on iodine intake. We have no data about the iodine supplementation status of our population, and we cannot exclude its role in TSH levels. However, since TSH variation determined slight Ctn variation, its role in clinical practice is marginal. In addition, the other parameters may be considered in specific cases in whom skewed Ctn cannot be explained according to gender and anthropometrics (e.g., men with small thyroid size). In any case, according to high-evidence data about ultrasound and FNAC (2, 20–22), careful management of patients with indeterminate Ctn must be focused on to avoid false negatives, and the next international guidelines are advised to prepare specific recommendations (25). Finally, companies producing Ctn assay are advised to develop clinically useful thresholds to help the decision-making process. Until then, the present results should be valid only when using the Ctn assays we used in populations with similar features to the present one from regions with comparable iodine supplementation status.

The strengths and limitations of the present study should be addressed. The present data were retrospective and were extracted from patients managed by several physicians over time. At our institution, no guidelines about testing Ctn have been implemented until 2022. A cost analysis was not feasible because the series was not consecutive. Two Ctn assays were used during the study period. In addition, the herein-reported population is smaller than that presented in other studies in this field. This is due to highly selective criteria included in the study design that were undertaken to achieve the most reliable series of cases with full (i.e., histological) exclusion of MTC. Thus, the sample size might not allow us to fully explore less prevalent features (e.g., PPI). However, a major strength of this study is that it is the first one including all the factors previously reported as influencing Ctn in a series of patients with histological exclusion of MTC after total thyroidectomy.

In conclusion, this study demonstrates that, after a full analysis of a population histologically proven as MTC-free, the Ctn value is influenced by gender, anthropometric/thyroid features, and CKD. Thyroidologists and endocrinologists first have to be aware of these figures to better manage their patients during clinical practice. Thyroid nodule guidelines should incorporate these concepts.

## Data availability statement

The raw data supporting the conclusions of this article will be made available by the authors, without undue reservation.

## Ethics statement

The studies involving humans were approved by Tessin Cantonal Ethics Committee. The studies were conducted in accordance with the local legislation and institutional requirements. The participants provided their written informed consent to participate in this study.

## Author contributions

PT: Conceptualization, Data curation, Formal analysis, Funding acquisition, Investigation, Methodology, Project administration, Resources, Supervision, Validation, Writing – original draft, Writing – review & editing. GP: Data curation, Visualization, Writing – review & editing. DC: Data curation, Visualization, Writing – review & editing. EG: Investigation, Writing – review & editing. TP: Visualization, Writing – review & editing. FF: Visualization, Writing – review & editing. PM: Visualization, Writing – review & editing. AP: Formal analysis, Methodology, Validation, Writing – review & editing. LR: Data curation, Formal analysis, Investigation, Methodology, Software, Validation, Writing – original draft, Writing – review & editing.
